# A Walk on the Wild Side: Genome Editing of Tuber-Bearing *Solanum bulbocastanum*

**DOI:** 10.3390/plants13071044

**Published:** 2024-04-08

**Authors:** Aristotelis Azariadis, Olga A. Andrzejczak, Frida M. Carlsen, Ida Westberg, Henrik Brinch-Pedersen, Bent L. Petersen, Kim H. Hebelstrup

**Affiliations:** 1 Section for Crop Genetics & Biotechnology, Department of Agroecology, Aarhus University, Flakkebjerg, 4200 Slagelse, Denmark; aristotelisazariadis@hotmail.com (A.A.); hbp@agro.au.dk (H.B.-P.); 2 Section for Plant Glycobiology, Department of Plant and Environmental Sciences, Copenhagen University, 1871 Frederiksberg C, Denmark; fmc@plen.ku.dk (F.M.C.); iwt@plen.ku.dk (I.W.); blp@plen.ku.dk (B.L.P.)

**Keywords:** de novo domestication, genome editing, wild potato, transgene free, protoplasts

## Abstract

*Solanum bulbocastanum* is a wild diploid tuber-bearing plant. We here demonstrate transgene-free genome editing of *S. bulbocastanum* protoplasts and regeneration of gene-edited plants. We use ribonucleoproteins, consisting of Cas9 and sgRNA, assembled in vitro, to target a gene belonging to the nitrate and peptide transporter family. Four different sgRNAs were designed and we observed efficiency in gene-editing in the protoplast pool between 8.5% and 12.4%. Twenty-one plants were re-generated from microcalli developed from individual protoplasts. In three of the plants we found that the target gene had been edited. Two of the edited plants had deletion mutations introduced into both alleles, whereas one only had a mutation in one of the alleles. Our work demonstrates that protocols for the transformation of *Solanum tuberosum* can be optimized to be applied to a wild *Solanum* species.

## 1. Introduction

The common cultivated potato (*Solanum tuberosum*) originated near the Titicaca Basin in southern Peru as documented by archaeological finds [1,2] and in agreement with genetic data [3]. However, several other tuberous *Solanum* species exist.

We here demonstrate gene editing and plant regeneration in the wild tuberous species *S. bulbocastanum*. There are several methods for transforming and genome editing of *S. tuberosum* [4,5,6]. But as noted by [7] such methods may be difficult to directly transfer to other tuber-bearing *Solanum* species. As of now there are no successful reports of genome editing in *S. bulbocastanum.* In this study, we establish the first protocol for genome editing of this species using transgene-free mediated gene editing and single protoplast cell regeneration to full plants and demonstrate its application through the introduction of loss-of-function mutations consisting of 2–8 bp deletions in a target gene. As a proof-of-concept for genome editing in *S. bulbocastanum* we choose a gene from the nitrate and peptide transporter family. This family represents a large group of transporters divided into eight clades (see [8] for a full review). Transporters belonging to this family have been shown to transport a huge diversity of specialized metabolites including: glucosinolates, cyanogenic glucosides, flavonol diglucosides, monoterpene indole alkaloids, and steroidal glycoalkaloids. They are involved in both cell import and cell export of specialized metabolites, as well as trans-membrane transportation within the cell. The function of the target gene in potatoes has not yet been described for any members of this family. However, *Solanum* species contain a high number of genes belonging to this family, making it an interesting target for gene function studies by targeted knock-out.

## 2. Materials and Methods

### 2.1. Plant Material

Plants of *S. tuberosum* var. Desireé were obtained from KMC (Brande, Denmark). In vitro grown plantlets of diploid *S. bulbocastanum* (WKS 35323) were obtained from IPK (Leibniz, Germany). Plantlets were propagated in a phytotron growth cabinet with 70% humidity, 16 h light/8 h dark with 24 °C/20 °C and a light intensity of 40–60 µmole m^−2^ s^−1^, as donor material for leaf protoplast as described in [9].

### 2.2. Design of sgRNAs

For the design of single guide RNAs (sgRNA), the program Benchling CRISPR assembly “https://www.benchling.com/crispr (accessed on 10 June 2021)” was used with the SL3.0 (*Solanum lycopersicum*) genome as input and ‘NGG’ (SpCas9, 3′ side) as PAM sequence. From there, sgRNAs with a high target score efficiency were selected (Supplementary Materials S1 and S2). Our gene of interest (GOI) was cloned from *S. bulbocastanum* using primers designed from the *S. tuberosum* genome (Forward: 5′-ATGGAGCAAGAGATGGCGGA-3′; reverse: 5′-CTAGCAGGAATTCAAGTCAGATG-3′). The gene itself is a potential membrane transporter of glycoalkaloids, whose homologue was previously found in tomato [10]. As neither the *S. bulbocastanum* nor *S. tuberosum* genomes are available in the Benchling database, the off-target score could not be determined. The detailed sequence can be found in Supplementary Materials S1.

### 2.3. Protoplast Isolation and Test for Viability

Isolation of protoplasts was initially based on the method described by [11]. Incubation times, macerozyme concentration, and sucrose concentration were modified. The optimized final protocol was as follows.: Leaves from 4–6-week-old in vitro grown plants were harvested and cut in thin slices (1–2 mm) in Petri dishes containing 45 mL of medium B (2.7 g/L MS, 0.01% vitamin solution, 100 mg/L casein hydrolysate, 2 mg/L NAA, 0.5 mg/L BAP, KOH to pH 5.8) the same day. After slicing, Medium B was replaced with plasmolysis solution (0.5 M D-sorbitol). The leaf slices were incubated for 20 min in the dark, at 25 °C without shaking. The plasmolysis solution was then replaced with medium C (including 0.3% macerozyme and 1% cellulase RS). The Petri dishes were incubated in the dark, overnight, at 25 °C without shaking. The following day, protoplasts were evicted from the cell walls by gently shaking for 1 h at room temperature. Protoplasts were then filtered through 100 and 70 µm pre-wetted filters, then washed in a wash solution. The protoplasts were centrifuged gently at 50× *g* for 5 min, with slow acceleration and deceleration, and the protoplast pellet was resuspended in a new wash solution. The resuspended protoplasts were placed in a centrifuge tube containing a 0.35 M sucrose solution. Following centrifugation at 50× *g* for 15 min, the intact protoplasts assembled in a thick dark band between the two solutions. Then, the protoplast layer (ca 3 mL) was transferred to 3 mL of transformation buffer 1 (190 mM mannitol, 100 mM CaCl_2_, 0.5% (*w*/*v*) MES, KOH to pH 5.6). The protoplasts in this solution were kept at 4 °C when the 20 µL of the cell solution was examined in a microscope. An addition of 0.4 µL of 5 mg/mL fluoresceine was used to identify intact living cells and counted in a hemocytometer.

### 2.4. Preparation of Ribonucleoproteins (RNPs) and Genome Editing of Protoplasts

For each transformation, RNPs were assembled by mixing 37.5 pmol of modified TrueGuide^TM^ sgRNA (Invitrogen, Waltham, MA, USA) with 37.5 pmol (2.5 µg) TrueCut^TM^ Cas9 v.2 (Invitrogen, Waltham, MA, USA), which resulted in the Cas9 being in a 1:1 ratio with the sgRNAs. The mix was incubated for 12–16 h at 4 °C. Freshly prepared protoplasts were gently centrifuged at 50× *g* for 10 min, then resuspended in transformation buffer 2 (500 mM mannitol, 15 mM MgCl_2_, 0.1% (*w*/*v*) MES, KOH to pH 5.6) to a final concentration of 1.6 × 10^6^ protoplasts/mL. The RNPs (10–20 µL of sgRNA-CAS complex) were then transferred to room temperature (appx. 25 °C) and gently mixed with 200 µL 1.6 × 10^6^ protoplasts/mL followed by an addition of 200 µL 25% *w*/*v* PEG solution. The transformation was stopped after 10 min by adding 5 mL of Wash solution to each tube. Protoplasts were edited by a mix of two different RNPs. We used the three combinations sgRNA1 and sgRNA3, sgRNA1 and sgRNA4 and sgRNA1 and sgRNA5 (Supplementary Materials S1 and S2). For RNP combinations, each RNP was incubated separately and then mixed just before transformation to a final concentration of 37.5 pmol of RNPs. The transformed protoplasts incubated with the RNPs were split into two parts. One was used for PCR mediated efficiency of editing analysis by Indel detection by amplicon analysis (IDAA) [12] as described below, the other half was used for regeneration of plants.

### 2.5. Indel Detection by Amplicon Analysis (IDAA) for Editing Efficiency in Protoplasts and Regenerated Plants

For editing efficiency in protoplast, protoplasts were washed with a wash buffer, centrifuged with 5000× *g* for 10 min, and dissolved in 50 µL of MiliQ water. Then, denaturation conditions were applied (95 °C for 10 min) and 5 µL of the solution was used for tri-primer PCR. For an analysis of regenerated plants, DNA was isolated using the FastDNA Plant DNA Kit (MP biomedicals, Irvine, CA, USA) according to the producer’s protocol. The quality and quantity of DNA was assessed using NanoDrop spectrophotometer and diluted to 50 ng/μL. Tri-primer PCR was performed with FAM labelled primers as specified in Supplementary Materials S2. PCR of the exons 1 and 2 was performed using PROFILase 2x Master Mix (COBO Technologies, Copenhagen, Denmark). Tri-primers (Supplementary Materials S2) set up was completed in 25 μL reaction with 50 ng genomic DNA or 5 µL of protoplasts. A touchdown program was used with pre-denaturation of 15 min at 95 °C, followed by 15 cycles with 95 °C, for 30 s, annealing 72 °C–1 °C/cycle for 30 s and extension 72 °C for 30 s, and 25 cycles with annealing at 58 °C for 30 s. The final extension was performed for 30 min in 72 °C. In total, 5 μL of PCR product was run on 3% agarose gel and stained in ethidium bromide for 25 min. The rest of the PCR product was sent to COBO technologies (Copenhagen, Denmark) for IDAA analysis.

Editing efficiency was calculated from the peak areas in the IDAA chromatogram using the online software ProfileIt 1.0 in VIKING https://viking-suite.com/ (accessed on 3 April 2023).

### 2.6. Regeneration of Plants from Cas9-gRNA RNP Edited Protoplasts

Protoplasts were embedded in alginate lenses (2.8% (*w*/*v*) alginic acid, 0.4 M D-sorbitol) floating in Petri dishes in liquid medium E which was changed every 3–4 days. The Petri dishes were incubated at 25 °C for 3 days in dark, then transferred to constant light (30 µmole m^−2^ s^−1^), while also being covered by 3 pieces of tissue paper to reduce the light intensity for the youngest protoplasts while initiating microcallus development. The tissues were removed one by one every 24 h, to reach the desired full light intensity (of 30 µmol m^−2^ s^−1^) at 6 days after embedding. Once the protoplast microcalli were visible to the naked eye (usually after 3 weeks), medium E was replaced with 10 mL medium F. Medium F was changed every 7 days. At day 50, post protoplast isolation (ppi) the microcalli, having reached a size of ~1–3 mm, were released from the alginate lenses by placing them in medium G (2.7 g/L MS, 267.5 mg/L NH_4_Cl, 0.1% vitamin solution, 80 mg/L adenine sulphate, 100 mg/L casein hydrolysate, 2.5 g/L sucrose, 36.4 g/L mannitol, 0.1 mg/L IAA, 2.5 mg/L zeatin, KOH to pH 5.8). Depending on the size of the microcalli, duration on medium G varied from 0 to 14 days. Calli large enough (≥3 mm) were moved directly to medium H, so medium G can be used as a recovery medium for weak or late-growth calli. Calli of at least 3 mm size and bright green in color, were then transferred to solid medium H for further callus growth and shoot regeneration and incubated at a diurnal cycle of 16 h light/8 h dark with 24 °C/20 °C and light intensity of 40–60 µmole m^−2^ s^−1^. When shoots were around 10 mm (about 90 days ppi) they were excised from the calli and transferred to plant propagation medium (PPM) (20 g/L sucrose, 0.1 mM kinetin, 4.3 g/L MS, NaOH to pH 5.8) and grown in the same conditions as above. The plants could be further propagated by cutting internodal stem sections and propagating these in the same medium. Fully developed plantlets appeared after approximately 4 weeks. Control plants from non-edited protoplasts were regenerated in parallel as well.

### 2.7. Genotyping by Sequencing

The same primers as for the IDAA analysis (without the FAM sequence—Supplementary Materials S2) were used to amplify exon 1 and 2 of the GOI. DNA was extracted using Fast DNA kit (MP biomedicals, Irvine, CA, USA) according to producer protocol. Touchdown PCR using Herculase II Fusion DNA Polymerase (Agilent, Santa Clara, CA, USA) was performed with 2 min of denaturing at 95 °C; 12 cycles of denaturing at 95 °C for 20 s; annealing at 70–1 °C per cycle for 20 s and extension 72 for 30 s; followed by 25 cycles of denaturing at 95 °C for 20 s; annealing at 58 °C per cycle for 20 s and extension 72 for 30 s; followed by final extension for 4 min. PCR products were run on a 1% agarose gel, and then isolated and purified from the gel with SPINeasy PCR Purification and Gel Extraction Kit (MP biomedicals, Irvine, CA, USA). For each PCR product, TOPO blunt end reaction was performed with Zero Blunt^TM^ TOPO^TM^ PCR Cloning Kit (Thermofisher, Waltham, MA, USA). Chemically competent Stellar *E. coli* cells (TakaraBio, Saint-Germain-en-Laye, France) were transformed with 2 μL of TOPO reaction and spread on the LB plates with white/blue screening. After overnight incubation at 37 °C, 5 colonies for each mutant were picked and inoculated in 3 mL LB medium. Plasmids were isolated using a Miniprep spin kit (QIANGEN, Venlo, The Netherlands) and sequenced using Sanger sequencing (Macrogen Europe, Amsterdam, The Netherlands) with universal M13 primers (forward and reverse). Analysis of the sequencing data was conducted using Benchling and consensus sequences were produced, which were then analyzed for similarities using ClustalOmega alignment.

## 3. Results

### 3.1. Optimizing Isolation of Leaf Protoplasts from S. bulbocastanum

**Figure 1:**
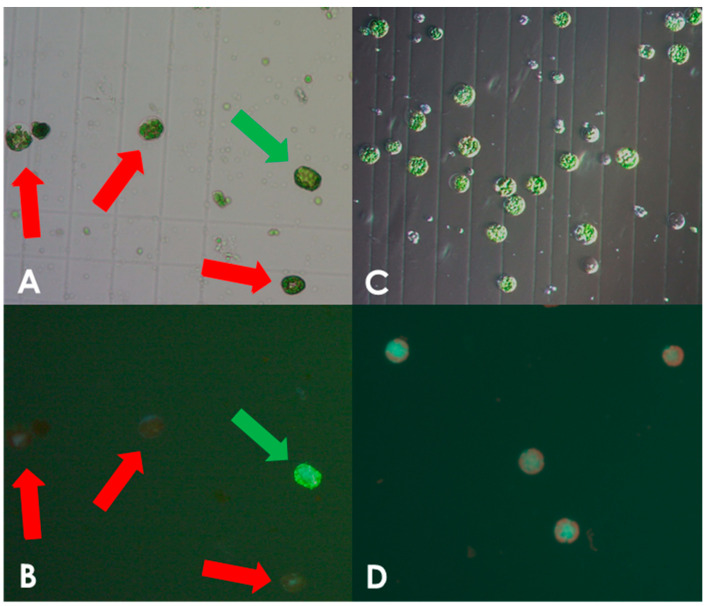
Optimization of protoplast isolation. Protoplast isolated using the non-optimized protocol for *S. tuberosum* showing viable and burst cells as indicated by green and red arrows, respectively, under white light (**A**) and fluorescein staining (**B**). Only very few viable *S. bulbocastanum* protoplasts could be isolated when using the protocol for *S. tuberosum*. Protoplast isolated after protocol adjustment, showing viable, spherical shapes with no debris present, under white light (**C**) and fluorescein staining (**D**).

Protoplasts isolated from leaves of in vitro grown *S. bulbocastanum* plantlets were transformed with in vitro assembled DNA-free ribonucleoprotein (RNP) complexes, consisting of the Cas9 enzyme and the sgRNA (Supplementary Materials S1 and S2) as described in the Section 2. We initially used protocols developed for potato (*S. tuberosum*), which however, yielded an insufficient number of intact viable protoplasts. When examining cells in the microscope we found that there was a high prevalence of burst protoplasts that showed traces of cell wall debris. This indicated incomplete cell wall digestion (Figure 1A,B). The concentration of macerozyme was optimized accordingly as described in the Section 2. In line with this, we noted that leaves of *S. bulbocastanum* are generally thicker, larger, and have a more robust appearance than those of *S. tuberosum*, because of this the incubation time for cell wall digestion was increased. Leaves were harvested and protoplast released the same day. Following enzymatic liberation from the dissected leaves, protoplasts were obtained from the interphase between a low-density medium on top of a high sucrose density solution. At this stage, we found that the majority of the cells had ruptured, indicating a possible imbalance in osmosis. Changing the concentration of mannitol in the buffer did not solve the problem. However, when adjusting the sucrose concentration of lower phase of high-density solution from 0.43 M to 0.35 M, a satisfactory number of viable protoplasts was obtained (Figure 1C,D). Following these optimizations we successfully obtained protoplasts. In three isolations, we obtained 6 mL of 1 × 10^6^ protoplast/mL, 0.750 × 10^6^ protoplasts/mL, and 1.25 × 10^6^ protoplasts/mL, respectively. As described in the Section 2, protoplasts were concentrated by centrifugation to a final concentration of 1.6 × 10^6^ protoplasts/mL and were used for genome editing.

### 3.2. Genome Editing of Protoplasts

**Figure 2:**
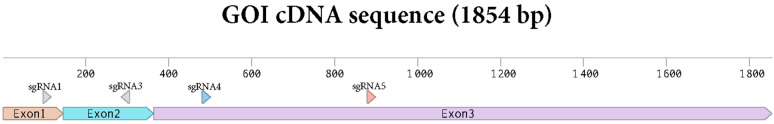
Sequence of the cDNA of the GOI with the sgRNAs targets depicted.

Protoplasts were edited by PEG infiltration with CRISPR/Cas9 RNPs as described in the Section 2. sgRNAs were designed to target exon 1 to 3 of the gene of interest (Figure 2). Two sgRNAs were applied in each editing experiment with the aim of creating deletions between the two sgRNA target sites. Genome editing efficiency at in the protoplast pool was estimated by isolating gDNA and performing a PCR amplification of the target area as described in the Section 2 and using the primers shown in Supplementary Materials S2.

**Figure 3:**
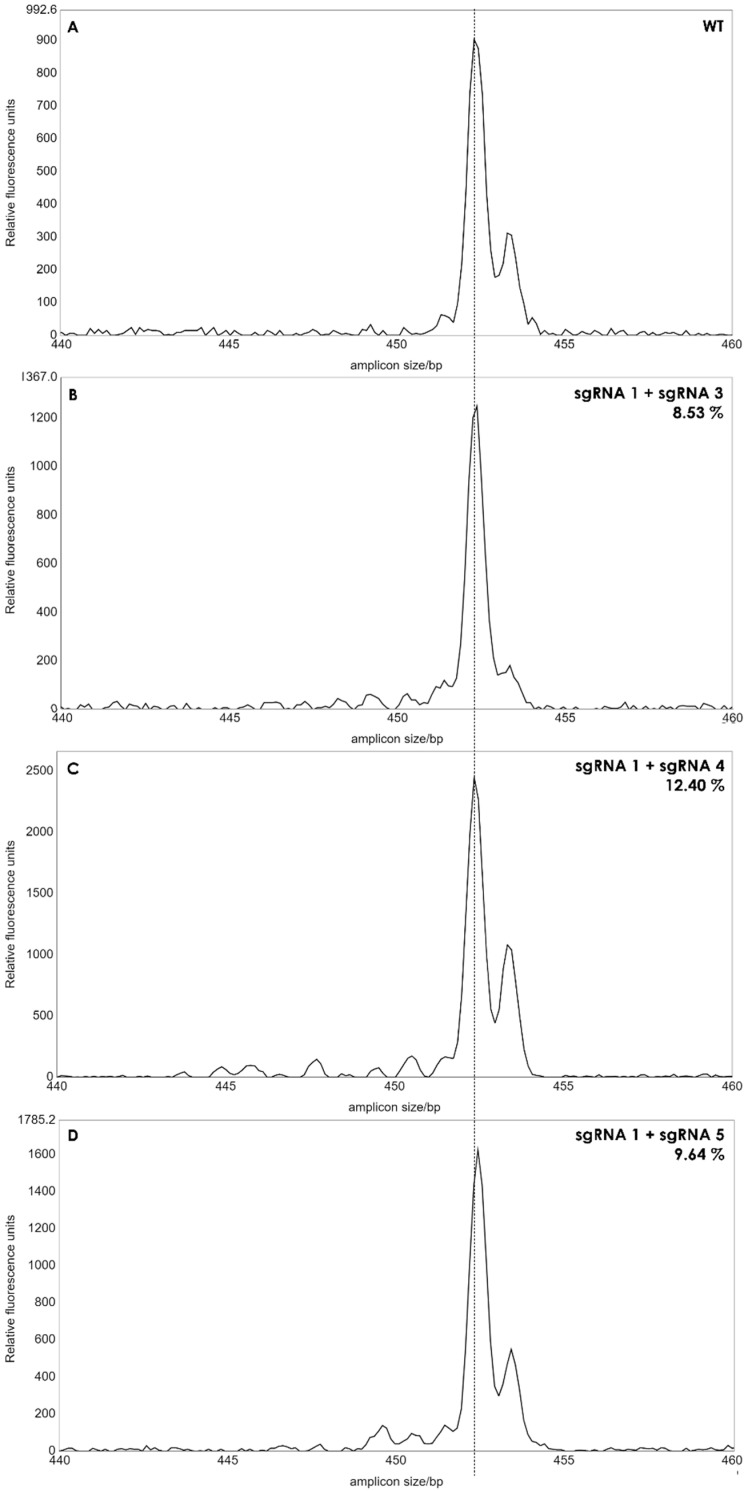
IDAA profiles of edited protoplast cells. The amplicon size of 453 bp of the wild type (WT) was used as reference (**A**). Small peaks from 440 to 452 bp’s indicated deletions between 1 to 13 bp in batches of protoplasts. The peak areas of the deletions for each sgRNA combination (**B**–**D**) was calculated as percentages, as described in [12]. Dashed line indicates amplicon size in WT plants.

A single band was observed for each reaction, and the length of the PCR products from the pool of protoplasts was similar to that of wild type plants, when analyzed in agarose gels (not shown) indicating that no larger deletions had occurred with a high frequency in the protoplasts. We then applied IDAA (Indel Detection by Amplicon Analysis), which is capable of separating DNA fragments with size differences down to +/−1 bp using capillary electrophoresis [12]. Editing efficiency can be calculated from the IDAA chromatograms as the area under the curve of tops corresponding to indels different from the expected amplicon of wildtype haplotypes [9]. The IDAA chromatograms from a wildtype control and the three pairwise combinations of sgRNAs is shown in Figure 3. We did not observe deletions that would correspond to fragment delineated simultaneously by two sgRNAs which would have been a deletion at the size of more than 100 bp. We observed only small deletions between 1 and 13 bp, (Figure 3B–D). The three different combinations of the sgRNAs showed different editing efficiencies: 8.53% for sgRNA1 and sgRNA3, 12.4% for sgRNA1 and sgRNA4 and 9.64% for sgRNA1 and sgRNA5 (Figure 3). It has been observed previously that the frequency of edited protoplast is a good indicator for the number of gene edited plants when plants are regenerated from the protoplast pool [9]. We, therefore, regenerated plants from each of the three editing experiments.

### 3.3. Regeneration of Plants from Edited Protoplasts

**Figure 4:**
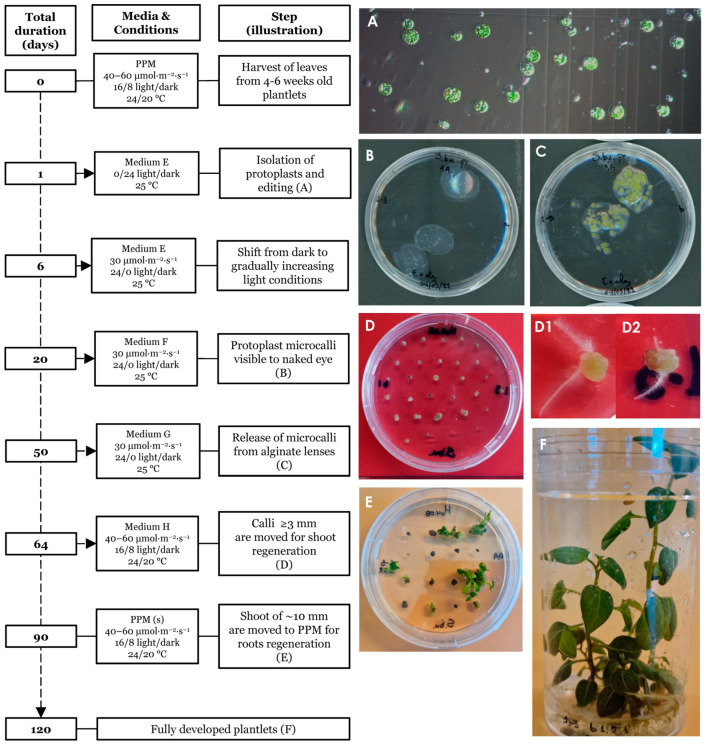
Timeline of the regeneration protocol. Isolated protoplasts prior to transformation (**A**). Day 20 post protoplast isolation (ppi) showing protoplast embedded in alginate lenses (**B**). Day 50 ppi, prior to release from the alginate lenses (**C**). Day 64 ppi, hardened calli showing initiation of early plant growth (**D**). Calli with premature root emergence (**D1**,**D2**). Day 90 ppi, presence of shoots of ~10–20 mm length (**E**). Day ~120 ppi, fully regenerated plants grown in propagating medium (**F**).

Plants were regenerated from edited single protoplasts as summarized and outlined in Figure 4. The protocol spans an approximately 120 days from isolation to fully regenerated plants. Isolated single protoplasts (Figure 4A) were embedded in alginate lenses (Figure 4B) and allowed to propagate clonally using the hormones NAA and BAP (medium E) to induce the formation of microcalli (Figure 4C). Individual microcalli were then released from the protective lenses and placed on solid agar plates containing the hormones NAA, zeatin and GA_3_ (medium H) for continued callus growth (Figure 4D). During this stage, some of the calli started to form hairy roots (Figure 4(D1,D2)). Individual calli were then transferred to shoot medium and finally to root/proliferation medium from where fully regenerated plants were obtained. Light intensity was adjusted for growth and the survival of regenerative tissues throughout the procedure as described in the Section 2. At the shoot regeneration stage (Figure 4E) many calli initially appeared senescent, taking a long time to generate shoots. We found that this process could be accelerated by cutting the apparently senescent calli in two and placing them with the freshly cut surface onto the medium, indicating that cell death had occurred at the exposed surface, reducing the cell regeneration potential and the core contained live cells with higher regeneration potential.

### 3.4. Confirmation of Gene Editing in Regenerated Plants by IDAA and Sanger Sequencing

**Figure 5:**
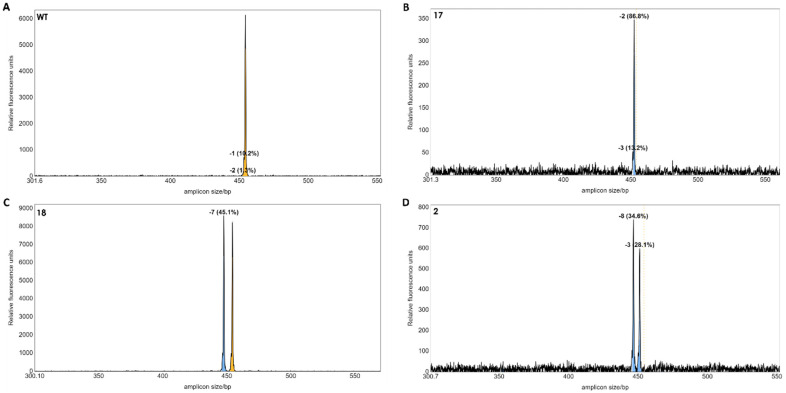
IDAA profiles of regenerated individual plants. The wild-type control showed a peak at the 453 bp of the two WT alleles (**A**). Mutant 17 appeared monoallelic with a 2 bp deletion in 86.8% PCR products (**B**). Mutant 18 appeared to be heterozygous with a deletion of 7 bp in a single allele (**C**). Mutant 2 appeared to be biallelic with deletions of −8 and −3 bp, respectively (**D**). Dashed line and yellow colour indicate the size of the WT gene. The blue colour indicates a deletion mutation.

A total of 21 regenerated plants from potentially edited single protoplasts were obtained. Genomic DNA was extracted from each plant, and the potential indels were detected by IDAA using the tri-primer amplification method according to [12]. IDAA is quantitative and, therefore, the area under the curve will correspond to the frequency of alleles with length polymorphism. This is particularly useful for polyploid species, such as tetraploid *S. tuberosum* [9]. But since *S. bulbocastanum* is diploid [13], two equally prominent peaks correspond to two alleles with length polymorphism. The IDAA suggested that three of the 21 plants had indel mutations in the target gene (Figure 5). Mutant line no 17 contained a homoallelic top corresponding to a 2 bp deletion (Figure 5B). Mutant line no 18 contained a 7 bp deletion in one allele and one wild type (WT) allele (Figure 5C). Mutant 2 appeared biallelic with two peaks corresponding to a 8 bp and a 3 bp deletion, respectively (Figure 5D). The mutant lines 17 and 18 were obtained from editing using sgRNA1 and sgRNA5. Mutant 2 was obtained from editing using sgRNA1 and sgRNA3.

**Figure 6:**
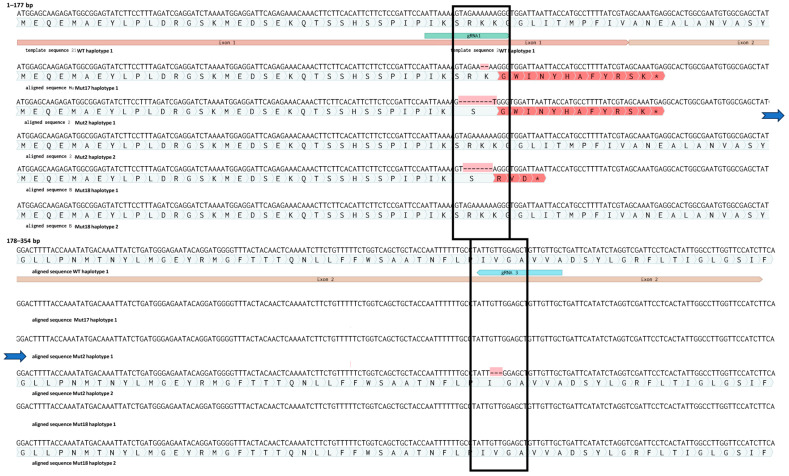
Mutations in haplotypes from edited plants 2, 17 and 18. Asterisk at the end of the red region indicates a premature stop codon (for details see Supplementary Materials S3). The blue arrows indicate a continuation of the sequence.

Sequencing of ten cloned amplicons from each mutant line confirmed the IDAA results (Figure 6). We identified one allele with a 2 bp deletion of two adenines (A’s) localised 3 bp upstream of the PAM site of sgRNA1 in all ten clones of mutant 17. This monoallelic homozygous mutation leads to a frameshift followed by a premature stop codon in the beginning of exon 2 (Supplementary Materials S3). Therefore, this plant is predicted to contain a full loss-of-function mutation in the gene of interest. Mutant 18 has a deletion of a −7 bp (AGAAAAA) sequence 4 bp upstream of the PAM site of sgRNA1 on one allele. The other allele appeared to be WT, and the amplicon genotype is therefore in agreement with the IDAA analysis, which suggests that the plant is heterozygous. This mutation also leads to a frameshift followed by a premature stop codon at the end of exon 1. Biallelic mutant 2 had a deletion of eight bp (AGAAAAAA) in 1 allele, 4 bp upstream of the sgRNA1 PAM site. This mutation also leads to a frameshift and a premature stop codon in the beginning of exon 2. However, the second allele of mutant plant 2 contained an in-frame deletion of 3 bp (GTT), which leads to a deletion of a single amino acid, a valine. The deletion in this allele is situated 4 bp in upstream of the sgRNA3 PAM site (Figure 6).

sgRNA1 conferred the majority of the RNP mediated mutations (Figure 6). sgRNA1 was also predicted to be the most efficient (Supplementary Materials S2). While sgRNA3 conferred the −3-deletion allele in mutant 2, neither sgRNA4 nor sgRNA5 conferred apparent mutations at the target sites in the regenerated plants highlighting the importance of designing several different gRNAs. The number of mutations induced by each of the sgRNAs correlated with their predicted efficiencies (Supplementary Materials S2). Regenerated edited plants were grown in vitro up to 1 month, at which point they were further propagated as described in the Section 2. None of the regenerated plants at that stage showed a morphological phenotype different to WT plants, whether being regenerated directly from protoplasts or from internodes of adult fully developed plants.

## 4. Discussion

We have established a protocol for transgene-free genome editing of wild *S. bulbocastanum*. Three separate protoplast editing experiments were conducted. In each case pairs of RNPs were co-transformed and IDAA analyses revealed protoplast editing efficiencies of 8.53%, 12.40% and 9.64%, respectively (Figure 3). In two *S. tuberosum* cultivars [9,14], protoplast editing efficiencies were found to be cultivar-dependent and in particular, the sgRNA in question. Knock out of a recessive gene/trait in tetraploid *S. tuberosum* cultivars requires generation of knock-out mutations in all four alleles in order to obtain a complete loss of gene activity. Theoretically, an editing efficiency of 50% in protoplasts should lead to 12.5% of plants with editing in all four alleles of a tetraploid plant if the events of editing in homologous alleles happens independently. However, ref. [9] found that plants with full knock-out exceed this theoretical level, suggesting that once RNP is inside the cell it will on the average confer editing of more than 1 of the 4 alleles in the cell. The protoplasts are likely to be in different stages of the cell cycle. In the mid-interphase between the mitotic pro-, meta-, ana- and telophases, the DNA is less condensed than during the mitotic cell division. It is possible that the DNA and chromatin may be more prone to editing in certain stages of the cell cycle. Since *S. bulbocastanum* is diploid, less editing efficiency may be needed to obtain plants with full homozygous or biallelic loss-of-gene function in a target gene. In this study, we regenerated 21 plants from protoplast and found that 2 of them had deletion mutations introduced into both alleles, whereas one only had a mutation in one of the alleles. This is in line with the observation of [9] that multiple editing events happen at a higher frequency than expected if mutations events were fully uncorrelated. It remains to be tested if multiplexing with RNPs targeting different genes also demonstrates a correlated frequency, and if the editing frequency that we have observed in *S. bulbocastanum* is enough to be able to conduct multiplexing. The intention behind the design of using two sgRNAs was to induce larger deletions. But we did not find haplotypes, where the two sgRNAs had induced mutations simultaneously in the same chromosome, either by inducing a larger deletion between their target sites or by inducing smaller deletions in each target site. It is possible that editing frequency needs to be higher to archive this. With three mutations induced by sgRNA1 and one mutation induced by sgRNA3, out of 21 regenerated plants the predicted sgRNA efficiency (Supplementary Materials S2) seems to be a good indicator of editing efficiency. It is possible that if two sgRNAs with an efficiency like that of sgRNA1 had been used, we would have observed lines with larger deletions between two sgRNAs.

Genome editing in potatoes has been carried out by different methods [4,5,6]. The CRISPR/Cas9 protein can be delivered through a genetic vector in the form of a plasmid DNA, brought into the plant cells by biolistic transformation or by PEG infiltration or it can be transferred as T-DNA from *Agrobacterium tumefaciens*. The cells may be in the form of callus in tissue culture or protoplasts. We here used the alternative method of transgene-free editing by applying RNPs directly to protoplast cells. This has some advantages over other methods. In the future, plants with transgene-free DNA deletions may be regulated in EU countries similar to that of plants generated by traditional breeding methods, such as chemical or physical mutagenesis. Whereas plants with transgenic elements, such as the CRISPR/Cas9 construct, are likely to still be still regulated under a GMO-like directive [15]. It is possible to remove the CRISPR/Cas9 construct by back-crossing or segregation in generations later than the T0. However, potato is usually vegetatively propagated and therefore back-crossing or propagation through seeds is likely to induce loss of heterosis. By editing protoplast and regenerating plants from single cells, rather than transforming callus, we also minimize the risk of getting plants with mosaic genotypes. This is particularly problematic in T0 plants [16]. Also, because this method does not imply the use of selection markers, edited plants can be reused for further editing, whereby stacking of new genotypes can be achieved. 

The method presented here does not include integration of transgenic elements into the genomic DNA. However, under current legislation any deletion, even single base-pair deletions induced by CRISPR/Cas9 are still considered GMO in several areas of the world including the EU. However, deletions of genomic DNA induced by genome editing may be regulated as conventional plants in the future within EU countries [15,17]. This protocol may also be adapted for genome editing of other wild *Solanum* species.

## Data Availability

Data are contained within the article and Supplementary Materials.

## Supplementary Materials

The following supporting information can be downloaded at: https://www.mdpi.com/article/10.3390/plants13071044/s1.

## Abbreviations

BAP—6-benzylaminopurine; EBN—endosperm balance numbers; FAM—carboxyfluorescein; GA20ox-1—Gibberellic acid 20 oxidase 1; GAME—glycoalkaloid associated metabolism; GOI—gene of interest; IDAA—indel detection by amplicon analysis; IAA—indole-3-acetic acid; MES—2-(N-morpholino)ethanesulfonic acid; MS medium—Murashige and Skoog medium; NAA—naphthaleneacetic acid; NBS-LRR—nucleotide-binding site and a leucine-rich repeat domain; ppi—post protoplast isolation; PPM—plant propagation medium; R-genes—resistance genes; RNP—ribonucleoprotein.
