# Supplementary material for: A Walk on the Wild Side: Genome Editing of Tuber-Bearing Solanum bulbocastanum
Source: Plants (Basel). 2024 Apr 8;13(7):1044. doi: 10.3390/plants13071044 (PMC11013279; doi:10.3390/plants13071044)
Supplement: Supplementary file 1 [file plants-13-01044-s001.zip › plants-2903002-supplementary.pdf]

Supplementary material

S1. Sequence of the cDNA of the gene of interest with the sgRNA target sequences depicted.

S2. Sequence of sgRNA used and the primers.

**sgRNA**					
**Name**	**Position**	**Strand**	**Sequence**	**PAM**	**Efficiency Score**
sgRNA1	114	1	ATTAAAAGTAGAAAAAAGGG	TGG	54.67
sgRNA3	289	-1	GCAACAACAGCTCCAACAAT	AGG	42.47
sgRNA4	498	1	TCGTTTCTGCTCTTGTCAAT	TGG	27.15
sgRNA5	896	1	TTCAGGACCTCAAGTGCCAA	CGG	43.81
**Primers used for IDAA**					
**Name**	**Type**	**Sequence**			**Concentration**
FAM	Universal FAM	5'-6-FAM-AGC TGA CCG GCA GCA AAA TTG-3'			0.25 µM
Trans22_F_1	For-extension forward	5' AGC TGA CCG GCA GCA AAA TTG GCA AGA GAT GGC GGA GTA TC 3'			0.025 µM
Trans22_R_2	Reverse	5' TGA AGA TGG AAC CAA GGC CA 3'			0.25 µM

S3. Alignment of the mutated alleles with coding sequences (**A**) and predicted amino acid sequences (**B**). Red amino acids indicate change in reading frame.

S4. Media and Stock solutions recipes modified from Nicolia et al.(2015)

**Medium PPM**	**1 liter**
MS Salts + vitamins	4.3 g
Sucrose	20 g
Kinetin (10 mM stock)	100 µl
Agar	3.5 g
pH	5.8

**Medium B**	**1 liter**
MS modif. No 4	2.7 g
Vitamins NN stock	0.1 ml
Casein hydrolysate	100 mg
NAA Soluble in EtOH	2 mg
BAP Soluble in MeOH	0.5 mg
pH	5.8

**Medium C**	**500 ml**
Macro stock	5 ml
CaCl2 stock (2 M)	1.5 ml
Iron stock	5 ml
Micro stock	500 µl
Vit mix 1 stock	2.5 ml
Vit mix 2 stock	2.5 ml
Vit mix 3 stock	2.5 ml
Sugars stock	10 ml
Organic acids stock	5 ml
Casein hydrolysate	250 mg
Glucose 0.2 M	18.5 g
Mannitol 0.2M	18.5 g
PVP 10	10 g
NAA Soluble in EtOH	0.5 mg
BAP Soluble in MeOH	0.2 mg
Cellulase RS	5 g
Macerozyme	1.5 g
pH	5.6

**Medium E**	**1 liter**
Macro stock	10 ml
$CaCl_2$ stock (2 M)	1.25 ml
Iron stock	10 ml
Micro stock	1 ml
Vit. Mix 1 stock	5 ml
Vit. Mix 2 stock	5 ml
Vit. Mix 3 stock	5 ml
Sugars stock	20 ml
Organic acids stock	10 ml

Casein hydrolysate	500 mg
Glucose	33,7 g
Mannitol	30,92 g
BSA	1 g
NAA	1 mg
BAP	0.4 mg
pH	5.6

**Medium F**	**1 liter**
MS modif. No 4	2,70 g
NH_4Cl	107 mg
Vit. NN stock	1 ml
Adenine sulphate	40 mg
Casein hydrolysate	100 mg
Sucrose	2.5 g
Mannitol	54,7 g
NAA	0.1 mg
BAP	0.5 mg
pH	5.8

**Medium G**	**1 liter**
MS modif. No 4	2,70 g
NH_4Cl	267.5 mg
Vit. NN stock	1 ml
Adenine (hemi)sulphate	80 mg
Casein hydrolysate	100 mg
Sucrose	2.5 g
Mannitol	36,4 g
IAA	0.1 mg
Zeatin	2.5 mg
pH	5.8

**Medium H**	**1 liter**
MS salts and organics	4,4 g
Sucrose	10 g
Vit. NN stock	1 ml
NAA	0.01 mg
Zeatin	2.0 mg
GA_3	0.1 mg
pH	5.8
Gelrite	2.5 g

**Plasmolysis solution**	**1 liter**
D-sorbitol	91.1 g / 0.5 M

**Wash solution***	**1 liter**
Macro stock	10 ml

CaCl2 stock (2 M)	3 ml
Iron stock	10 ml
Micro stock	1 ml
NaCl	14.03 g
NAA	2 mg
BAP	0.5 mg
pH	5.6

**Sucrose solution**	**1 liter**
Sucrose	119.8 g

**Transformation buffer 1**	**500 ml**
Mannitol	17.3 g
$CaCl_2 * 2H_2O$	7.34 g
MES	2.5 g
pH	5.6

**Transformation buffer 2**	**500 ml**
Mannitol	45.5 g
$MgCl_2 * 6H_2O$	1.52 g
MES	500 mg
pH	5.6

**PEG solution**	**100 ml**
PEG 4000	25g
Mannitol stock (0.8 M)	50 ml
$Ca(NO_3)_2$ stock (2 M)	5 ml

**Alginate solution**	**1 liter**
Alginic acid-Na salt	28 g
D-sorbitol	72.88 g

**Setting agar**	**1 liter**
Sorbitol	72.88 g
$CaCl_2 * 2H_2O$	7,351 g
Phyto agar	8 g

**Floating solution**	**1 liter**
Sorbitol	72.88 g
$CaCl_2 * 2H_2O$	7,351 g

**Releasing solution**	**1 liter**
Na-citrate	5.88 g
Sorbitol	91.1 g

Stocks solutions

**Fluorescein stock**	**1 ml**
Fluoresceine diacetate	5 mg
Acetone	1 ml

**Macro stock**	**1 liter**
KNO_3	74 g
$MgSO_4$, $7H_2O$	49,2 g
KH_2PO_4	3,4 g
Store at 4°C	

**Iron stock**	**100 ml**
Na_2EDTA	140 mg
$FeSO_4$, $7H_2O$	190 mg
Store at 4°C	

**Micro stock**	**100 ml**
H_3BO_3	150 mg
$MnSO_4$ * H_2O	500 mg
$ZnSO_4$ * $7H_2O$	100 mg
Na_2MoO_4 * $2H_2O$	12 mg
$CuSO_4$ * $5H_2O$	1,2 mg
$CoCl_2$ * $6H_2O$	1.2 mg
KI	38 mg
Store at 4°C	

**Vitamins NN stock**	**50 ml**
Glycine	100 mg
Myo-Inositol	5000 mg
Thiamine-HCl	25 mg
Pyridoxine-HCl	25 mg
Nicotinic acid	250 mg
Folic acid	25 mg
Biotin	2.5 mg
Store at -20°C	

**Vit. mix 1 stock**	**100 ml**
Pantothenic acid	50 mg
Choline chloride	50 mg
Ascorbic acid	100 mg
p-Aminobenzoic acid	1ml
Nicotinic acid	50 mg
Pyridoxine-HCl (Vitamin B6)	50 mg
Thiamine-HCl	500 mg
Store at -20°C	

**Vit. mix 2 stock**	**100 ml**
Folic acid	20 mg
Biotin – H1 vit	0.5 mg
Cyanocobalamin – B12 vitamin	1 mg
Store at -20°C	

**Vit. mix 3 stock**	**100 ml**
Cholecalciferol – D3 vitamin	0.5 mg
Store at -20°C	

**Sugars stock**	**100 ml**
Sorbitol	625 mg
Sucrose	625 mg
D(-)Fructose	625 mg
D(-)Ribose	625 mg
D(+)Xylose	625 mg
D(+)Mannose	625 mg
L(+)Rhamnose monohydrate	625 mg
D(+)Cellobiose	625 mg
Myo-Inositol	250 mg
Store at 4°C	

**Organic acids stock**	**100 ml**
Pyruvic acid	100 mg
Fumaric acid	200 mg
Citric acid monohydrate	200 mg
L-Malic acid	200 mg
Store at 4°C	

GOI cDNA sequence (1854 bp)

(from 1-856 bp)

```
ATGGAGCAAGAGATGGCGGAGTATCTTCCTTTAGATCGAGGATCTAAAACGGAGGATTCAGAGAAGCAAACTTCTTCACATTCTTCTCCGATTCCAATTAAAAGTAG
TACCTCGTTCTCTACCGCCTCATAGAAGGAAATCTAGCTCCTAGATTTTGCCTCCTAAGTCTCTTCGTTTGAAGAAGTGTAAGAAGAGGCTAAGGTTAATTTTCATC
```

gRNA_1

Exon1

10 20 30 40 50 60 70 80 90 100

```
AAAAAAGGGTGGATTTATTACCATGCCTTTTATCATAGCAAATGAGGCACTGGAGAGTGTGGCGAGCTATGGACTTTTACCAAATATGACAAATTATCTGATGGGAC
TTTTTTCCCACCTAAATAATGGTACGGAAAATAGTATCGTTTACTCCGTGACCTCTCACACCGCTCGATACCTGAAAATGGTTTATACTGTTTAATAGACTACCCTG
```

Exon1 Exon2

110 120 130 140 150 160 170 180 190 200 210

```
AATACAGGATGGGGTTTACTACTGCTCAAAATCTTCTGTTTTTCTGGTCAGCTACTACCAATTTTTTGCCTATTGTTGGAGCTGTTGTTGCTGATTCATATTTAGGT
TTATGTCCTACCCCAAATGATGACGAGTTTTAGAAGACAAAAAGACCAGTCGATGATGGTTAAAAAACGGATAACAACCTCGACAACAACGACTAAGTATAAATCCA
```

gRNA_3

Exon2

220 230 240 250 260 270 280 290 300 310 320

```
CGATTCCTTACTATTGGCCTTGGTTCCATCTTCAGTTTCCTGGGATCAACAGTGTTGTGGTTAACAGCAATGATTCCGAAAGCCAGGCCTCCGCCTTGCAATCAAAC
GCTAAGGAATGATAACCGGAACCAAGGTAGAAGTCAAAGGACCCTAGTTGTCACAACACCAATTGTCGTTACTAAGGCTTTCGGTCCGGAGGCGGAACGTTAGTTTG
```

Exon2 Exon3

330 340 350 360 370 380 390 400 410 420

```
AGGACAGGCTTGTAATTCTACAACGGGACCACAATACATGCTCTTGGTTTTCTCGTTTCTGCTCTTGTCAATTGGTGCTGGAGGTATAAGACCATGTTCTTTAGCCT
TCCTGTCCGAACATTAAGATGTTGCCCTGGTGTTATGTACGAGAACCAAAAGAGCAAAGACGAGAACAGTTAACCACGACCTCCATATTCTGGTACAAGAAATCGGA
```

gRNA_4

Exon3

430 440 450 460 470 480 490 500 510 520 530

```
TTGGTGCAAACCAGTTTGACAAGGGAGGTAGCGATCCCAACAAACAGACAGTGTTGGAGAGCTTCTTTGCCTGGTATTATACTTCATCTGTAGTCTCTGTTCTGATT
AACCACGTTTGGTCAAACTGTTCCCTCCATCGCTAGGGTTGTTTGTCTGTCACAACCTCTCGAAGAAACGGACCATAATATGAAGTAGACATCAGAGACAAGACTAA
```

Exon3

540 550 560 570 580 590 600 610 620 630 640

```
GCCCTAACGGGTATCGTTTACCTTCAAGACAGACTTGGGTGGAAAATAGGTTTTGGAGTTCCTGCAATTCTCATGTTCTTATCCGCGTTGTTTTTCTTCCTTGCTTC
CGGGATTGCCCATAGCAAATGGAAGTTCTGTCTGAACCCACCTTTTATCCAAAACCTCAAGGACGTTAAGAGTACAAGAATAGGCGCAACAAAAAGAAGGAACGAAG
```

Exon3

650 660 670 680 690 700 710 720 730 740

```
TCCGTTTTATATCAAGCCAAAGGTTCGCTCAAATGTGTTTGCCAGCTTTATACGAGTAATTGTGGTTGCCTTCAAGAATAGGAAACTACATTACCCCAATCAGAACT
AGGCAAAATATAGTTCGGTTTCCAAGCGAGTTTACACAAACGGTCGAAATATGCTCATTAACACCAACGGAAGTTCTTATCCTTTGATGTAATGGGGTTAGTCTTGA
```

Exon3

750 760 770 780 790 800 810 820 830 840 850

GOI cDNA sequence (1854 bp) (from 857-1712 bp)

```
CTGATTATCATCACAAGAATGGTTCAGGACCTCAAGTGCCAACGGAGAAATTGAGATTCTTAAACAAAGCTTGCATCATTAAAAGCCCTGAAGATGTTAATCCAAAC
GACTAATAGTAGTGTTCTTACCAAGTCCTGGAGTTCACGGTTGCCTCTTTAACTCTAAGAATTTGTTTCGAACGTAGTAATTTTCGGGACTTCTACAATTAGGTTTG
```

gRNA_5

Exon3

860 870 880 890 900 910 920 930 940 950 960

```
GGAGTTGCAGCCAATCCATGGAACCTTTGCACAGTGGAGCAAGTTGAGGAGCTAAAAGCCCTCGTTAGAATCGTGCCATTGTGGTCAACAGGGATCATGATATCAAT
CCTCAACGTCGGTTAGGTACCTTGGAAACGTGTCACCTCGTTCAACTCCTCGATTTTCGGGAGCAATCTTAGCACGGTAACACCAGTTGTCCCTAGTACTATAGTTA
```

Exon3

970 980 990 1,000 1,010 1,020 1,030 1,040 1,050 1,060 1,070

```
AAACTTGAGCCAAAGTTCATTCCCACTACTACAAGCTCAATCCATGAATAGACATCTAACTAAAGGATTCCAAATTCCAGCAGGGTCATTCGGGATGTTTTTGATGA
TTTGAACTCGGTTTCAAGTAAGGGTGATGATGTTCGAGTTAGGTACTTATCTGTAGATTGATTTCCTAAGGTTTAAGGTCGTCCCAGTAAGCCCTACAAAAACTACT
```

Exon3

1,080 1,090 1,100 1,110 1,120 1,130 1,140 1,150 1,160 1,170

```
TTGCATTAACAATTTGGGTATTACTGTATGACCGCGTGATGCTTCCATTGGCATCAAAGATCAAAGGAAGACCAGTTCGTCTAAAACCTATAGTCAGAATGGGACTT
AACGTAATTGTTAAACCCATAATGACATACTGGCGCACTACGAAGGTAACCGTAGTTTCTAGTTTCCTTCTGGTCAAGCAGATTTTGGATATCAGTCTTACCCTGAA
```

Exon3

1,180 1,190 1,200 1,210 1,220 1,230 1,240 1,250 1,260 1,270 1,280

```
GGTATATTCGTGTCTTGCATGTCCATGGTAGTCTCTGGTATTATCGAAAATATTCGACGAAGAAGAGCAATCAGTGAAGGGCTGTTGAACAACTCGCAGGGGTTGGT
CCATATAAGCACAGAACGTACAGGTACCATCAGAGACCATAATAGCTTTTATAAGCTGCTTCTTCTCGTTAGTCACTTCCCGACAACTTGTTGAGCGTCCCCAACCA
```

Exon3

1,290 1,300 1,310 1,320 1,330 1,340 1,350 1,360 1,370 1,380 1,390

```
GGAGATGTCAGCAATGTGGCTCATTATACCAAACAGTTTAAACGGTATAGCAGAGGCGTTGAACGCGATTGGCGCCACAGAGTTCTATTATTCAGAGCTCCCAAAGA
CCTCTACAGTCGTTACACCGAGTAATATGGTTTGTCAAATTTGCCATATCGTCTCCGCAACTTGCGCTAACCGCGGTGTCTCAAGATAATAAGTCTCGAGGGTTTCT
```

Exon3

1,400 1,410 1,420 1,430 1,440 1,450 1,460 1,470 1,480 1,490

```
GTATGTCAAGTATTGCATCAGCTCTTTTAGGACTGGGAATGGCAGTTGCAAATCTTTTAGCAAGTGTTGTTTTGAGTGCTGTGGATAAGTACACGAAAGGAGAAGGG
CATACAGTTCATAACGTAGTCGAGAAAATCCTGACCCTTACCGTCAACGTTTAGAAAATCGTTCACAACAAAACTCACGACACCTATTCATGTGCTTTCCTCTTCCC
```

Exon3

1,500 1,510 1,520 1,530 1,540 1,550 1,560 1,570 1,580 1,590 1,600

```
AAAGAAAGTTGGATTTCAAGCAATATCAACAGGGGACACTATGAGTATTACTACTGGCTTCTTGCTCTAATGACAGGTTTTAATCTGCTTTATTTTGTGGTTTGTTG
TTTCTTTCAACCTAAAGTTCGTTATAGTTGTCCCCTGTGATACTCATAATGATGACCGAAGAACGAGATTACTGTCCAAAATTAGACGAAATAAAACACCAAACAAC
```

Exon3

1,610 1,620 1,630 1,640 1,650 1,660 1,670 1,680 1,690 1,700 1,710

GOI cDNA sequence (1854 bp) (from 1713-1854 bp)

```
CTGGCAATATGGACCTTCTGTTGATGTTGACATCACTATGAGAATGATGGAACCTAGCGACGATGAAGATGACGATGACGATGAAGATAAAGATGATTTGCCTAAGA
GACCGTTATACCTGGAAGACAACTACAACTGTAGTGATACTCTTACTACCTTGGATCGCTGCTACTTCTACTGCTACTGCTACTTCTATTTCTACTAAACGGATTCT
```

Exon3

1,720 1,730 1,740 1,750 1,760 1,770 1,780 1,790 1,800 1,810

```
AGAATAAATCTACACCTGACTTGAATTCCTGCTAG
TCTTATTTAGATGTGGACTGAACTTAAGGACGATC
```

Exon3

1,820 1,830 1,840 1,850

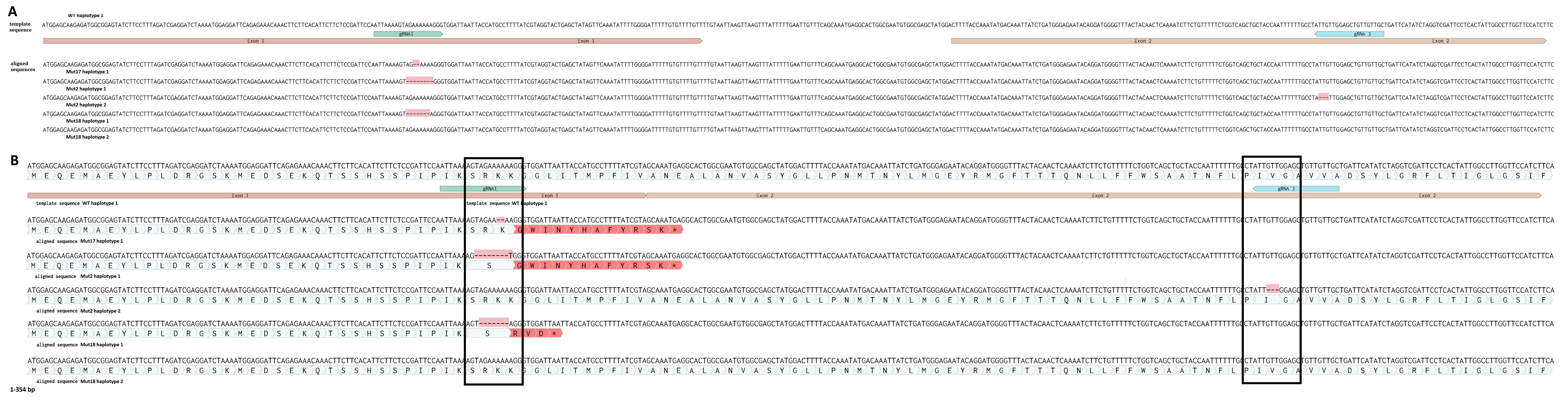
A
WT haplotype 1
template sequence
gRNA1
gRNA 3
Exon 1
Exon 2
aligned sequences
Mut17 haplotype 1
Mut2 haplotype 1
Mut2 haplotype 2
Mut18 haplotype 1
Mut18 haplotype 2
B
template sequence WT haplotype 1
aligned sequence Mut17 haplotype 1
aligned sequence Mut2 haplotype 1
aligned sequence Mut2 haplotype 2
aligned sequence Mut18 haplotype 1
aligned sequence Mut18 haplotype 2
1-354 bp
